# Failure Analysis of Tension Clamps (SKL15) Used in Serviced Urban Railway Tracks: Numerical Analyses and Experiments

**DOI:** 10.3390/ma15186354

**Published:** 2022-09-13

**Authors:** Jung-Youl Choi, Sun-Hee Kim, Sang-Jin Kim, Jee-Seung Chung

**Affiliations:** 1 Department of Construction Engineering, Dongyang University, No. 145 Dongyangdae-ro, Punggi-eup, Yeongju-si 36040, Korea; 2 Department of Architectural Engineering, Gachon University, 1342 Seongnamdaero, Sujeong-gu, Seongnam-si 13120, Korea; 3 Track Engineering Office 1, Seoul Metro, No. 5, Hyoryeong-ro, Seocho-gu, Seoul 06693, Korea

**Keywords:** tension clamp, Young’s modulus, middle band, laboratory test

## Abstract

In this study, the material properties of the damage-vulnerable parts and the residual strain of tension clamps comprising many curved parts, such as those used in urban railroads, were tested and analyzed. The effects of decreasing the strengths of the tension clamps on performance were then assessed. The permanent deformation characteristics of the tension clamps of aged specimens (6, 11, and 16 years of service) exhibited tendencies similar to the strain-hardening characteristics of the stress–strain responses reported in previous studies. As the service period increased, plastic deformation occurred in the middle bands of the tension clamps. When used for 16 years in urban railroads, the tension clamps underwent ~10% deformation compared with their initial shapes. Furthermore, based on laboratory tests, the deterioration levels of the tension clamps according to the service period were examined as functions of Young’s modulus. Stress levels close to the yield strength occurred in the middle band of the tension clamp when the clamping force was introduced. As a results, it is possible to determine whether the clamping force confirms that decrease by using the Young’s moduli of tension clamps, and the deterioration of the function and the replacement time of tension clamps that may occur during service can be predicted.

## 1. Introduction

**Figure 1:**
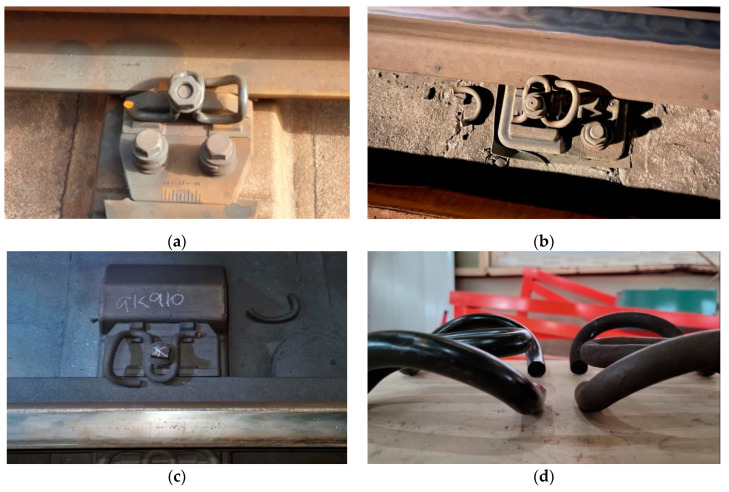
Images of damaged elastic rail fastening systems. (**a**) SKL12; (**b**) SKL14; (**c**) SKL15; (**d**) SKL15 deformation.

The quality of concrete tracks is affected by the role and performance of the elastic rail fastening system that simultaneously binds rails and sleepers and absorbs operation impacts directly. The urban railroads in South Korea operate over ballasted tracks, but to improve maintenance efficiency and track quality, the ballasted tracks were changed to concrete tracks in 2003, and an elastic rail fastening system was applied. The elastic rail fastening system experiences various types of damage when the service period increases to more than 20 years, as shown in Figure 1. In particular, damage caused by the breakage and deformation of tension clamps, which serve as fasteners, occurs frequently.

The core components of the elastic rail fastening system are tension clamps and elastic pads. Many studies have been conducted on elastic pads following the development of materials. Moreover, the establishment of replacement standards based on service life prediction and durability improvement is still being researched. However, studies have been primarily focused on elastic pads, whereas research on the required performance level, performance degradation, and proper maintenance plan for tension clamps has been insufficient.

Previous studies reported that tension clamps were used for sharp curve tracks, and there were many cases in which the stress was concentrated on the spring arm, which was damaged at the bent part. There were many cases of permanent deformation without breaking. Furthermore, Baik [1] found that the cause of the damage to the tension clamps was the uplifting force caused by the tilting of the sharp curved rail and demonstrated that 99% of the damage occurred in the bent part of the spring arm that received the greatest resistance to the uplifting force. Subsequently, another study [2] compared and analyzed the fatigue performance of broken tension clamps and new products. Moreover, they found that fatigue cracks could occur because the amplitude of the average stress that acted on the broken tension clamps exceeded the allowable fatigue stress. In addition, an increased initial strain occurred in the bent part of the spring arm during clamping force introduction. The behavioral characteristics when the initial strain was partially relieved due to the compressive deformation of the elastic pad because of a train load were also analyzed.

In a study on the improvement of the fatigue strength of tension clamps, Cho et al. [3] confirmed that plastic deformation occurred when the clamping force was removed and showed that the sensitivity to fatigue was high because stress was generated at levels close to the yield strength when the initial clamping force was introduced. Park et al. [4] reported that the surfaces of the tension clamps considered had decarburized layers with reduced carbon contents. When the depths of the decarburized layers of tension clamps with fatigue cracks were measured, they exceeded the maximum allowable depth (0.2 mm). The fatigue strength of the decarburized layer could cause fatigue cracking because it was lower than that of the base material. Kim et al. [5] developed a statistical model for the stress range by identifying the cause of the fracture of tension clamps and measuring the strain at the site of the largest fracture. They suggested the need for periodic inspection for fatigue damage because the reliability index of tension clamps decreased to values less than half of the initial reliability within 10 years. Park et al. [6] conducted a fatigue analysis of tension clamp fatigue cracks in a rail fastening system based on field measurements and finite element analysis. The results indicated the possibility of fatigue cracking because the stress amplitude of the tension clamps was larger than the allowable stress amplitude. To measure the deterioration level of coil spring ribs (e2007) due to long-term service, Seoul Metro [7] tested the clamping forces in four straight and four curved (R400m, R600m) sections in 12 products used for 25 years. The results suggested that the clamping force decreased with time, and the uplifting force caused by rail tilting could affect the decrease in the clamping force. Szurgott [8] evaluated the total deflections and stress states of the rail pads and the load distributions of all sleepers by performing numerical analyses of selected phenomena that occurred in a rail fastening system. Liu et al. [9] conducted a tensile strength test to derive the stress–strain responses of tension clamps and determined their strain hardening properties. El-Sayed [10] investigated the lateral load transmission mechanism based on a three-dimensional (3D) finite element analysis of concrete sleepers and a fastening system wherein vertical and lateral loads were combined. The findings indicated that the concrete–rail pad interface was influenced significantly by the high lateral-to-vertical loading ratio, which produced a localized contact area of high compressive stress. Xiao et al. used finite element (FE) analysis to evaluate the performance and predict the fatigue damage life of the e-clip under cyclic loading. The results confirmed that the inside of the rear arch has the shortest fatigue life and that it is therefore the most critical part [11].

In this study, we examined the elastic rail fastening systems used in actual lines from sections with 6 years, 11 years, and 16 years of service and performed a laboratory test under critical loading conditions by simulating a curve load. In addition, we derived the behavioral characteristics of tension clamps in curves, residual strain, causes of damage, and weak points, using numerical analyses. Furthermore, we experimentally confirmed the level of the material strength deterioration of tension clamps according to the service period and examined its effects on the performance of the tension clamps.

## 2. Tension Clamp

Tension clamps comprise spring steel and enable rail-fastener–sleeper binding. They are the core components that resist compression, levitation, and rail tilting, undergoing constant deformation during train running. The structures of tension clamps are the most affected by fatigue and are expected to undergo permanent deformation owing to long-term service.

**Figure 2:**
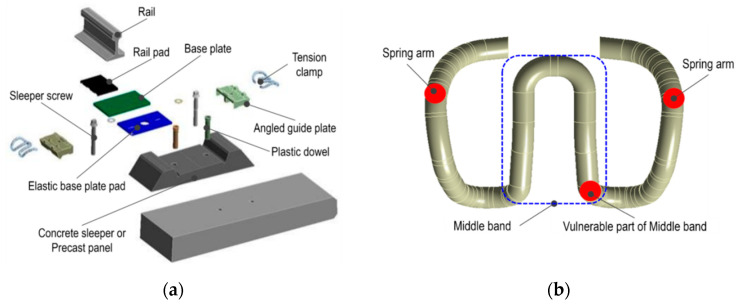
Schematic of elastic rail fastening system. (**a**) Components of elastic rail fastening system. (**b**) Main parts of tension clamp.

The components of the elastic rail fastening system, shown in Figure 2a, are composed of cold or hot rolled spring steel, as shown in Figure 2b. They have W-shapes with two spring arms that directly support the rails at both sides and a band on the center. The middle band is loaded by the rotational torque of the screw spike and is displaced in the vertical direction. At this time, the spring arms on both sides introduce clamping force to the rail. Tension clamps are prestressed structures and are used with a given initial clamping force. The clamping force of the tension clamps in contact with the rail reduces as the displacement of the elastic pad occurs when the train runs. When the load is removed, the uplifting force is transmitted to both arms, owing to the elastic restoration of the elastic pad, whereas the additional stress is transmitted to the tension clamps.

**Figure 3:**
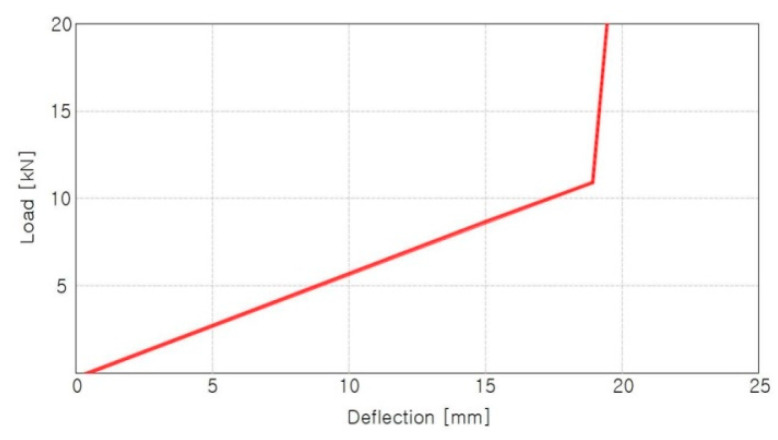
Load–deflection diagram for tension clamp (SKL15).

Figure 3 shows a load–deflection diagram of tension clamps. If a torque is applied without an initial load, the middle band is compressed, and the compressive load is transmitted to both spring arms. The optimum tightening torque used to introduce the clamping force is approximately 250 N·m. When a new product is tightened with the optimum tightening torque, the displacement of the middle band occurs at approximately 17 mm, and a clamping force in the range of 9.3–10.3 kN exists.

The deflection range of the elastic pad of the elastic rail fastening system is in the range of 0.6–0.8 mm when the tension clamps are first tightened. When a train passes, the secondary load is transmitted, resulting in a total displacement in the range of 1.2–1.6 mm. Tension clamps are members that always bear the fatigue load together with the elastic pads. Unlike elastic pads, which have relatively large areas, tension clamps have a very large stress generated in a very small cross-sectional areas (ø15 mm). Therefore, the effects on the fatigue are expected to be larger in the latter case. Moreover, the maintenance of the clamping force is crucial because tension clamps should play the most important role in fixing the rail position.

## 3. Laboratory Tests

### 3.1. Overview

**Figure 4:**
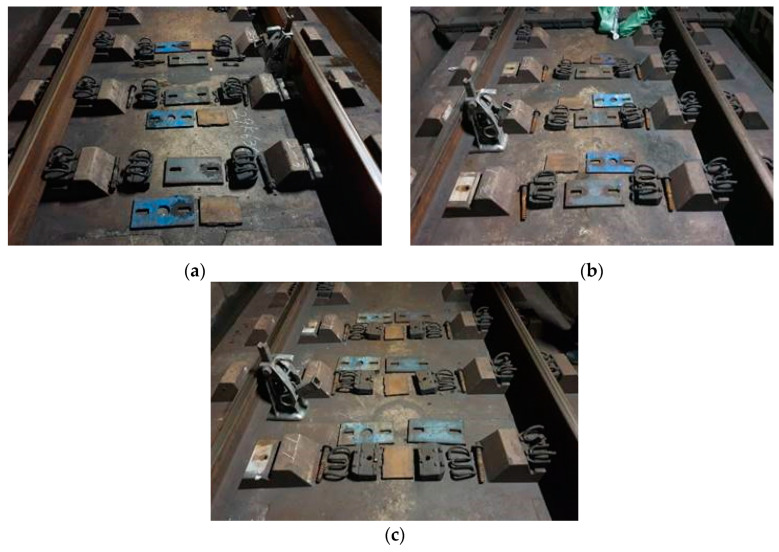
Collected specimens. Service period of (**a**) 6 years; (**b**) 11 years; (**c**) 16 years.

The standard for the number of repetitions of the fatigue test for railroad products is 3 million. The specimens used for 16 years in this study were the specimens that received a fatigue effect equivalent to 30 times the number of repetitions of the railway product performance test standard. Since it is fatigued 5,625,000 times per year, even in the case of a 6-year specimen, it has a fatigue effect (33,750,000 times = 33.75 million times) exceeding the current standard. The specimen exceeds the number of fatigue tests performed at laboratory scale. Since it is a specimen having a fatigue effect under actual operating load conditions, reliable data can be obtained even if the number of samples is small. For the specimen collection, three sites were selected with the same track type, rail, and geometric conditions, with service periods approximately equal to 6 years, 11 years, and 16 years. Table 1 lists the track specifications. The cumulative passing tonnage was calculated based on the calculation standards applied in the operation sections. The cumulative passing count for each section shows that the track was sufficiently loaded cyclically. Images of the specimen collection sites are provided in Figure 4.

### 3.2. Loading Test with an Ultimate Load on Curved Part

For the laboratory tests, the specimens were subjected to the inclination test conditions (type C, α = 33°) specified for cyclic loading tests by the Korean Railway Standards [7]. Specimens with service periods of 6, 11, and 16 years and a new product were tested six, respectively. In addition, strain gauges were attached to both ends of the spring arm of the tension clamp and the center of the middle band to measure the deflection.

**Figure 5:**
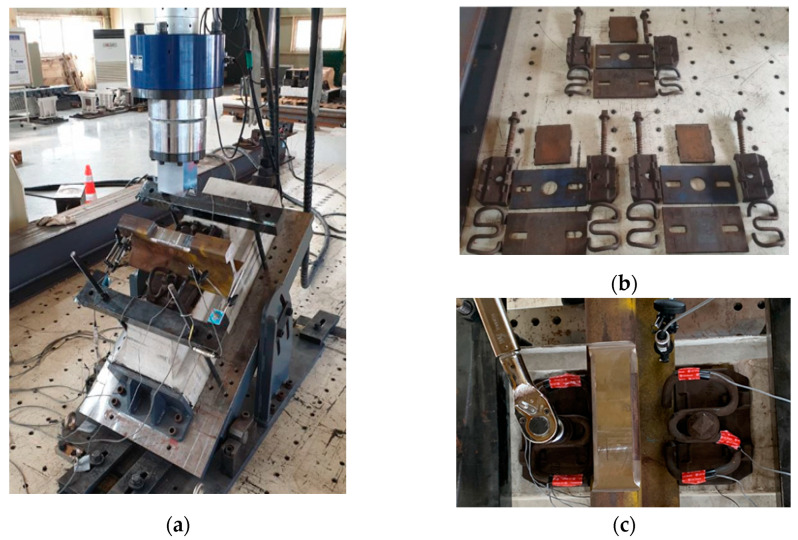
Images of laboratory test. (**a**) Inclination test; (**b**) specimen with 16 years of service; (**c**) introduction of clamping force.

The ultimate loading test was conducted by setting the magnitude of the load to 200 kN, which is larger than the test load of the normal rail fastening system. Figure 5a provides a photograph of the inclination test setting used for considering the curve load. Figure 5b shows the elastic rail fastening system collected from a section with 16 years of service. Figure 5c shows the introduction of the initial clamping force to the tension clamps. Regarding the test method, the first ultimate load was applied after tightening the tension clamps with the rated torque (250 N·m) by using a torque wrench. After the first load had been completely removed, the second ultimate load was applied. This process was repeated three times for each specimen.

### 3.3. Deformation Analysis of Middle Band Tension Clamp

In the tension clamps, the largest deflection deformation occurred in the middle band instead of the two arms. The reason that the large deflection occurs in the middle band is caused by cyclic load, and the resonance of the middle band is caused by the tension clamp corrugation. To determine the level of deformation of the middle band, the height of the middle-band height was measured according to the number of service years based on the new standard middle-band height and was found to be 42.5 ± 3 mm (mean ± standard deviation, range: 39.5–45.5 mm).

**Figure 6:**
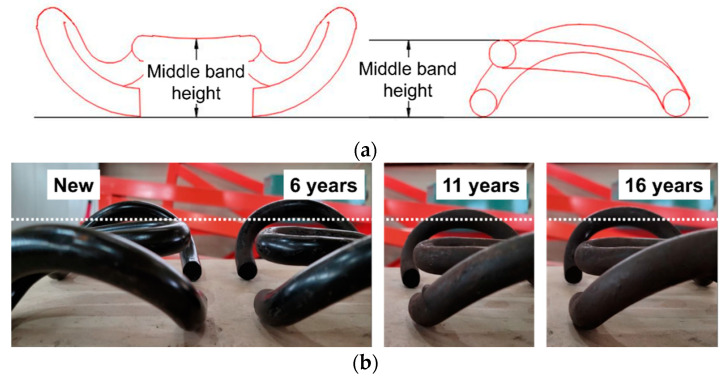
Schematic of deformation measurement for a middle-band tension clamp. (**a**) Measuring position of the middle-band height; (**b**) Deformation of the middle-band tension clamp according to the number of service years.

At the measurement position shown in Figure 6a, Gaussian probability density analysis was performed by measuring a total of six specimens for each service period with a vernier caliper three times for each service period, and the residual strain tendency was analyzed. Figure 6b shows the height deformation of the middle-band tension clamps according to the service period.

**Figure 7:**
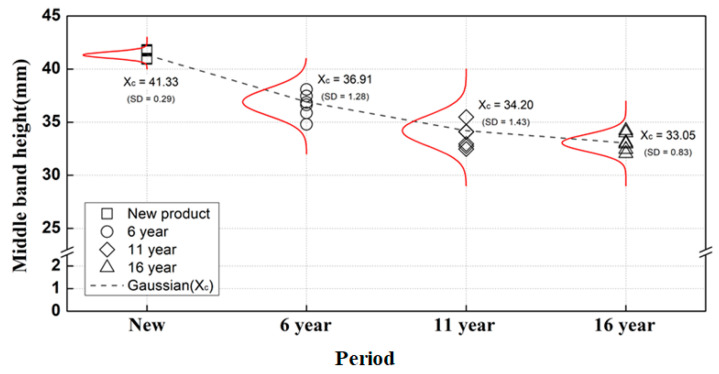
Measured residual strain of the middle band according to the service period for tension clamps.

The average and standard deviation of the probability density function were calculated by modeling the service period and displacement measurement results for each specimen as a normal distribution, as shown in Figure 7.

The standard deviation of the measured results based on the service period was calculated to be 0.29 for new products and 1.28, 1.43, and 0.83 for products at the 6th, 11th, and 16th years of service, respectively. The standard deviations for the tension clamps used for 6 to 11 years are increased compared to that for the new clamps. The deformations of the middle bands of the tension clamps are approximately 0.74 mm/year, 0.54 mm/year, and 0.23 mm/year in the specimens with 6, 11, and 16 years of service, respectively. The tension clamps appear to behave in the elastic region for up to 11 years. However, the yield point may be exceeded if the stress generated during use is increased because a stress close to the yield stress is imposed when the initial clamping force is introduced. Therefore, the height of the middle band was considered to be permanently reduced as residual strain (ε_s_ = 0.00839) generated after the yield point was accumulated, which can be attributed to the fact that the strain rate was maintained without an increase in stress after the occurrence of the residual strain rate at the yield point, as shown in the stress–strain diagram (38Si7 hot-rolled spring steel) derived from previous studies [1,2,3].

**Figure 8:**
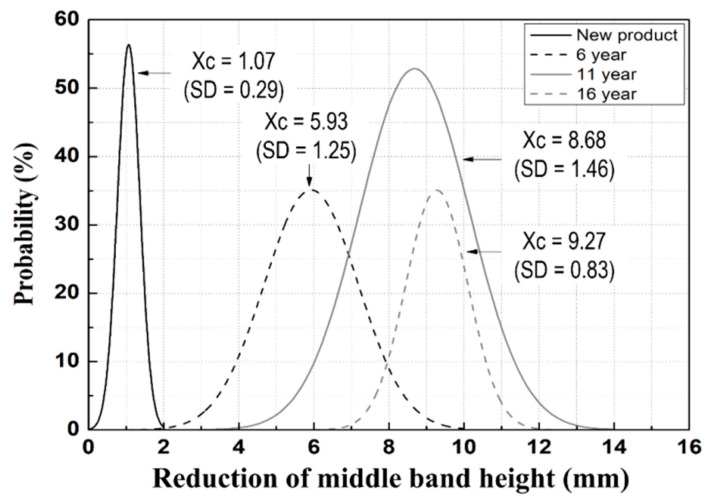
Reduction in the middle-band height of tension clamps.

Figure 8 presents the obtained height reductions of the middle bands of the tension clamps. The average decrease is 1.07 mm, 5.93 mm, 8.68 mm, and 9.27 mm for the new products and for those with 6, 11, and 16 years of service, respectively. Therefore, it was determined that the amount of reduction (93.7%) from the initial stage to the 11th year of service was dominant, and that the amount of reduction decreased after 11 years.

## 4. Numerical Analysis

### 4.1. Modeling

**Figure 9:**
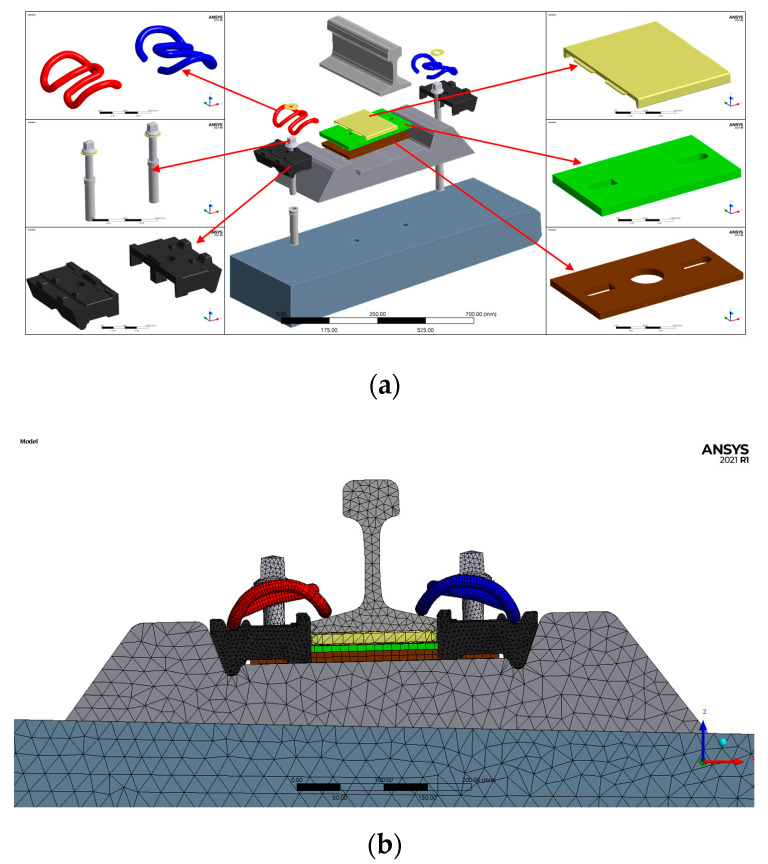

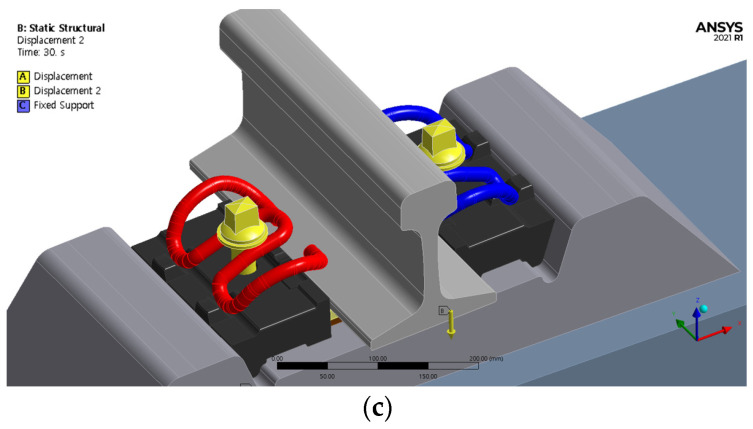
Three-dimensional modeling and boundary conditions. (**a**) Detail of FEA modeling; (**b**) detailed front (mesh); (**c**) screw spike displacement.

ANSYS Ver. 2021.1R (Canonsburg, PA, USA) was used as the finite element analysis program, and solid element was used for 3D modeling. The rails, tension clamp (SKL15), concrete panel, rail pad, resilience pad, guide plate, and base plate are composed of solid elements, as shown in Figure 9a. The mesh of the elastic rail fastening system model contained 369,871 nodes and 219,033 elements. For the boundary conditions, the lower part of the base plate was fixed under supported conditions, as shown in Figure 9b [12]. The rail clamping force was applied as a displacement to the screw spike, as shown in Figure 9c. The analyzed material properties are summarized in Table 2 and Table 3 [13]. For the load, cyclic load was applied to the upper part of the analysis model, similar to the experimental conditions.

**Figure 10:**
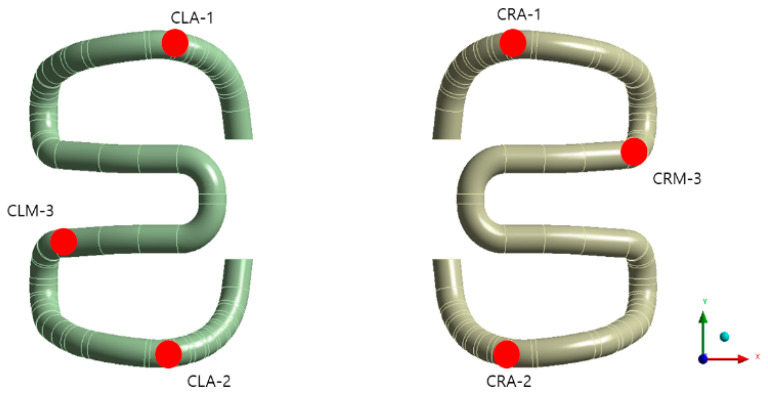
Positions defined for stress evaluation.

The numerical analysis results were calculated at the same position as that at which the strain gauge was installed on the tension clamp during the laboratory test, as shown in Figure 10.

**Figure 11:**
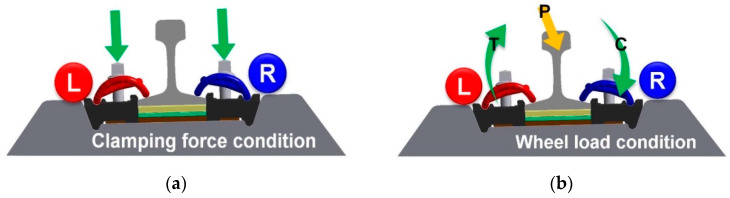
Conceptual diagram of the loading action of tension clamp. (**a**) Application of clamping force. (**b**) Application of curved track force.

As shown in Figure 11, when the elastic rail fastening system was secured, the same compressive stress was generated in the middle band and both arms of the inner (left) and outer parts (right of the gauge). Figure 11b is a conceptual diagram demonstrating the uplifting force that may act on the inner part of the gauge and the compressive force that may act on the outer part of the gauge due to the tilting of the rail by the loading action of the curved part.

Regarding the loading conditions, a load of 150 kN was applied, which is the resultant force attributed to the weight of the wheel (107.2 kN) and lateral pressure (89.4 kN). Furthermore, by applying the load–displacement diagram (clamping force diagram) for tension clamps, the displacement of the middle band (17 mm) was applied for displacement control when the initial clamping force was introduced.

**Figure 12:**
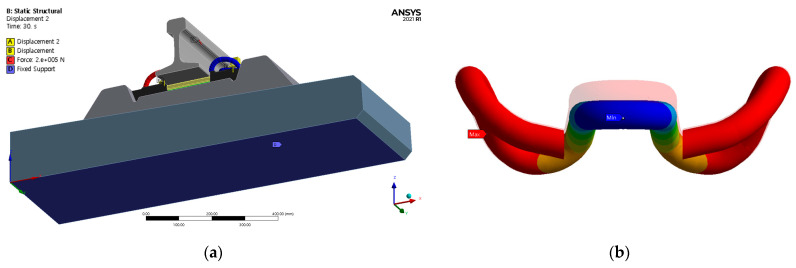
Overview of loads applied to rail and tension clamp. (**a**) Loading conditions. (**b**) Displacement control considering clamping force.

Figure 12a shows the directions and positions of the loads acting on the rails and tension clamps. Figure 12b shows the displacement control of the tension clamp subjected to loading.

### 4.2. Numerical Analysis Results

**Figure 13:**
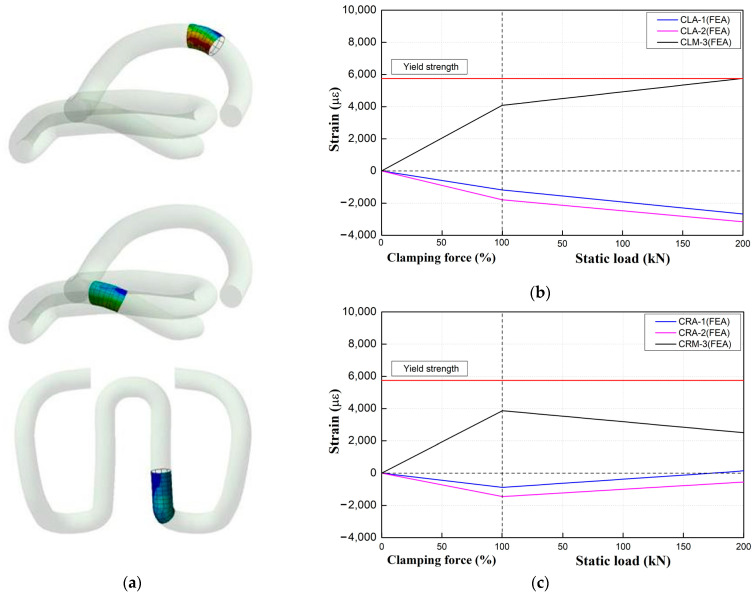
Numerical analysis results for tension clamps. (**a**) Stress-concentrated part. (**b**) Left strain. (**c**) Right strain.

Figure 13 shows the measured changes corresponding to the strains of the tension clamps inside and outside the gauge with each test load that simulated the loading condition of the curved part from the onset of clamping force introduction. The position at which the strain is maximized during clamping force introduction lies in the middle band. As the clamping force increases, the strain increases steeply, and the external force is managed until the yield strength limit is reached. Furthermore, the strain of the middle band occurs at the same level inside and outside the gauge.

When the clamping force is introduced, the strain generated in the middle band is close to the critical value. The strain rate of the middle band of the gauge outside the (right) tension clamps decreases according to the external load action, but the strain of the middle band inside the gauge increases; its value exceeds the critical value when the applied load is 200 kN. The compressive displacement of the elastic pad occurs when a compressive force is applied to the outer part of the gauge, owing to the tilting behavior of the rail according to the test load (which simulates the load on the curved part). In this process, the clamping force, first introduced to the spring arms of the tension clamps, is partially decreased, in turn decreasing the strain. In contrast, tensile strain is generated inside the gauge, owing to the upward displacement of the rail.

## 5. Analysis and Discussion

### 5.1. Analysis of the Changes of Young’s Moduli of Tension Clamps According to the Years of Service

**Figure 14:**
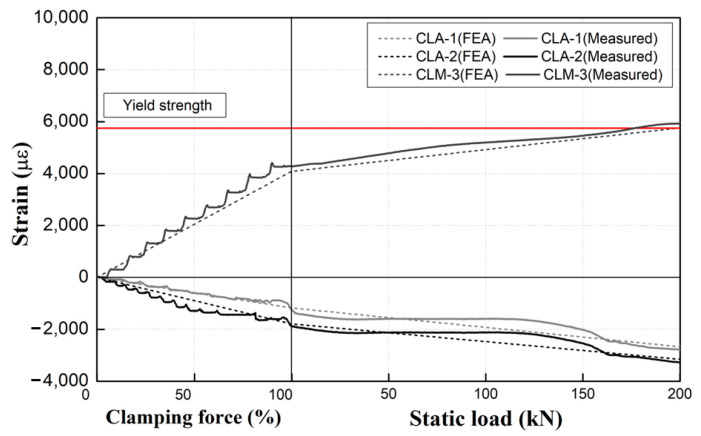
Comparison of experimental and analytical results for a new tension clamp.

The experimental and analytical results for a new tension clamp are presented in Figure 14. The two sets of results match closely during both the clamping force introduction and the external load application stage. Therefore, the numerical model employed in this study correctly reflected the behavioral characteristics of actual tension clamps.

**Figure 15:**
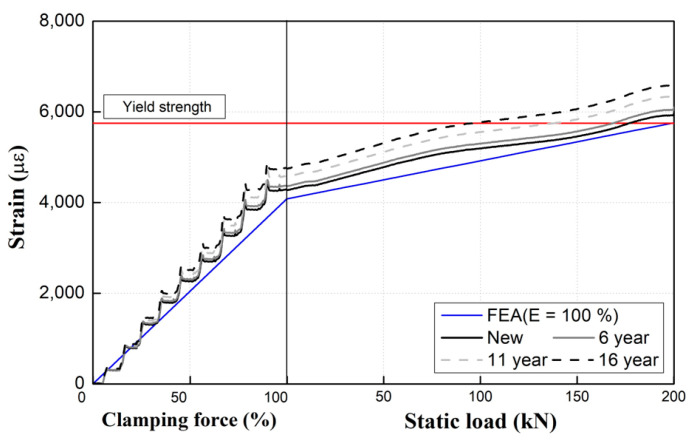
Comparison of experimental and analytical results for used products (E-100%).

However, as observed in Figure 15, the experimental results for used products (with service periods of 6, 11, and 16 years) are slightly different from the analytical results based on a Young’s modulus of 100% for new tension clamp products. Furthermore, as the specimen usage period increases, the difference between the analytical and experimental results increases as well. As shown in Figure 15, the critical value of the middle band for each specimen decreases as a function of the service period. In the case of the 16-year specimen, this value is decreased by approximately 50% compared with that of the 6-year specimen. Moreover, the numerical results obtained by using a Young’s modulus of 100% for the tension clamps show some deviation from the experimental results for each service period for the specimens subjected to the fatigue effect in the field. In particular, an increased difference in the strain gradient is evident from the clamping force introduction stage, which increases the deviation from the test results even during the loading stage.

In addition, as the service period increases, the deviation between the analytical and experimental results becomes larger, and the analyzed results overestimate the actual behavioral responses of the tension clamps with more than 6 years of service.

**Figure 16:**
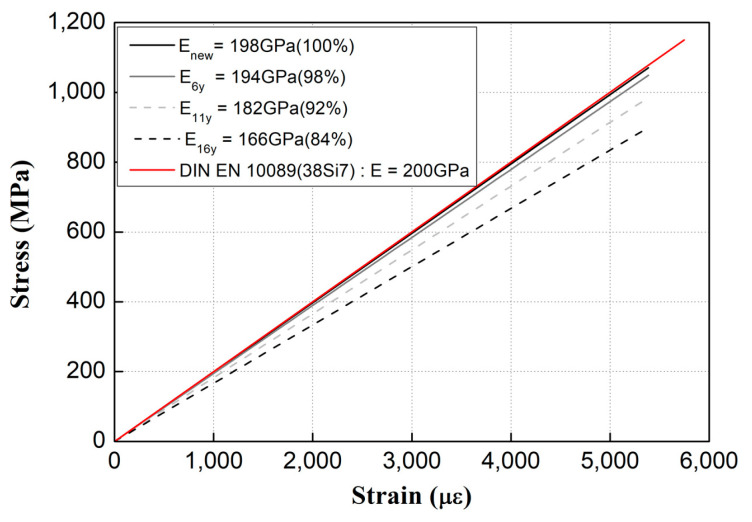
Stress–strain plots showing the variations of the Young’s moduli of the tension clamps.

Figure 16 presents the Young’s modulus according to the service period of tension clamps calculated based on the experimental results. As the service period increases, the Young’s modulus for the old products that have undergone plastic deformation decreses compared with that of the new product. Specifically, the Young’s modulus decreases by approximately 2%, 8%, and 16% for the 6-, 11-, and 16-year specimens, respectively.

### 5.2. Comparison between Analytical and Experimental Results Considering Young’s Modulus Changes for Tension Clamps

**Figure 17:**
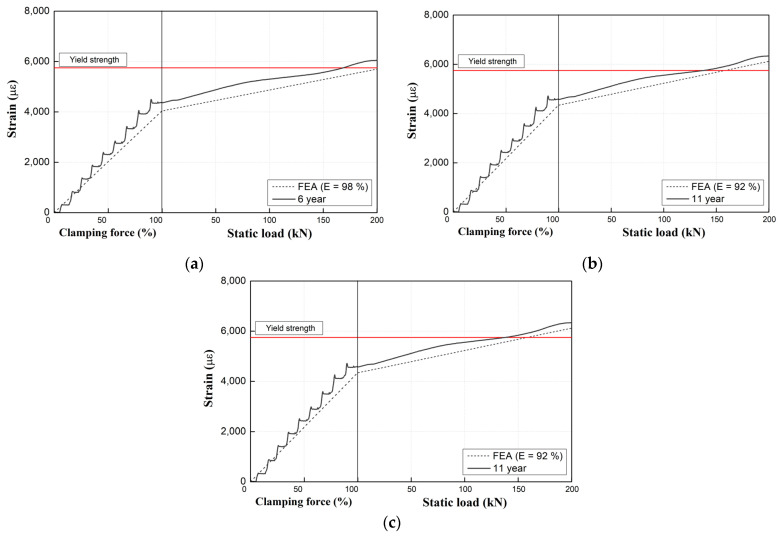
Comparison of analytical results with the measured Young’s moduli of tension clamps. Service period of (**a**) 6 years; (**b**) 11 years; (**c**) 16 years.

Figure 17 shows numerical analysis results obtained by using the Young’s moduli of aged tension clamps. The Young’s moduli of the tension clamps decreases with increasing usage period, but the maximum change rate up to 11 years is only 8%. Therefore, according to the results of this study, the change in the Young’s modulus of an aged tension clamp is minimal for up to 11 years or until the cumulative passing tonnage is reached. Thus, there are no significant effects on the function or performance of tension clamps. Further, it is appropriate to perform numerical analysis by applying Young’s modulus considering the actual service period to obtain reliable analysis results.

### 5.3. Analysis of Clamping Force Changes According to Tension Clamp Deterioration

**Figure 18:**
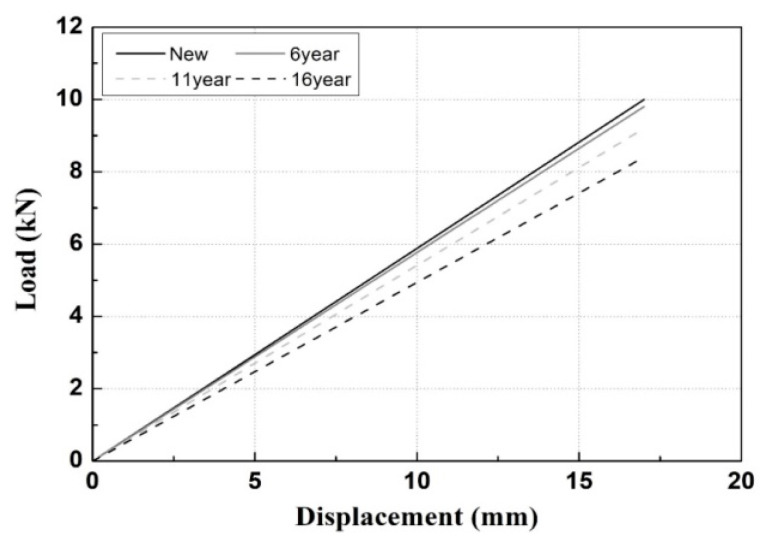
Load-displacement diagrams of new and used tension clamps.

Figure 18 depicts the load–displacement diagram obtained by numerical analysis and the use of the Young’s modulus for each service period. Evidently, the clamping force is approximately 10.0 kN, 9.8 kN, and 9.2 kN for new products, products with 6 years of service, and products with 11 years of service, respectively. The clamping force of the product with 16 years of service is decreased considerably in value and is approximately 8.4 kN.

The decrease in the Young’s modulus of the tension clamps implies decreases in the clamping force and resistance to the tilting of the rail. The slope of the load–displacement diagram indicates the stiffness of the member, whereas the external examination results show that the 24 tension clamps used in the experiment have no cross-sectional losses. Therefore, Young’s modulus directly affects the decrease in the stiffness of the tension clamps.

## 6. Conclusions

In this study, we experimentally and analytically derived the characteristics of the damage-vulnerable parts and the residual strains of tension clamps (which included many curved parts) used in urban railroads. The conclusions inferred from the analysis of the effects of the decreased strength of the tension clamps on the performance are as follows.

The investigation of the deformation levels of aged tension clamps using field specimens revealed that the middle-band height gradually decreased as the service period increased. A deformation of 19.92% on average occurred in products with 16 years of service compared with new products, confirming that residual strain could be induced by fatigue in the middle bands of tension clamps according to the service period.

The results of the investigation of the behavior of tension clamps at critical loads, and the weakening effect of damage showed that a stress close to the yield strength occurred in the middle bands of the tension clamps when a clamping force was introduced. Stress exceeding the yield strength can be generated in the middle band when the rail is tilted and when the resulting uplifting force is applied during service.

The Young’s moduli of the tension clamps did not change considerably until the 11th year of service. However, after 16 years of service, Young’s modulus and clamping force decreased by approximately 16%, and the critical load decreased by approximately 50%. Therefore, as it is possible to determine whether the clamping force is decreased by using the Young’s modulus of tension clamps, the deterioration of the function and the replacement time of tension clamps that may occur during service can be predicted.

Based on this study, the Young’s modulus, which describes the material strength of tension clamps, may decrease when the clamps have been used for a certain period. Furthermore, it can cause permanent deformation and decreases the clamping force.

The actual behavior may be overestimated when applying the Young’s moduli of new products in numerical analysis to evaluate the damage of aged tension clamps. Therefore, numerical analysis that uses the Young’s moduli of old products is appropriate when predicting the behavior of tension clamps and evaluating the load resistance performance (clamping force).

The inner and outer parts of the gauges of tension clamps may have dissmilar levels of deterioration because they have different applied load characteristics. Therefore, for urban railroads with many curved sections, it is necessary to establish a separate management plan for the tension clamps inside the gauge where the uplifting force acts and for those outside the gauge, where the downward force acts.

## Figures and Tables

**Table 1:** Parameters for the finite element model.

Number	Years of Service	Cumulative Passing Tonnage (ton)	Cumulative Passing Count	Annual Passing Count	Speed (km/h)
1	16 years	550,802,913	89,523,250	5,595,250	60
2	11 years	364,011,979	61,547,250		70
3	6 years	181,637,388	33,571,500		80

**Table 2:** Material properties of the finite element model.

Component	Young’s Modulus (MPa)	Poisson’s Ratio (ʋ)	Density (kg/m^3^)
Rail	2.0 × 10^5^	0.30	7850
Tension clamp (SKL15)	2.0 × 10^5^	0.26	7800
Concrete panel	34,000	0.18	2500
Rail pad	24.5	0.30	950
Resilience pad	19.5	0.49	850
Guide plate	8500	0.40	1150
Base plate	1.73 × 10^5^	0.30	7850

**Table 3:** Material properties of the tension clamp (38Si7).

Yield strength (MPa)	1150
Tensile strength (MPa)	1300–1600
Young’s modulus (GPa)	200–207
Poisson’s ratio (ʋ)	0.27–0.30
Density (kg/m^3^)	7850

## Data Availability

Data sharing is no applicable to this article.
